# Lamprey immune protein triggers the ferroptosis pathway during zebrafish embryonic development

**DOI:** 10.1186/s12964-022-00933-0

**Published:** 2022-08-17

**Authors:** Zeyu Du, Duo Zhang, Jun Li, Qingwei Li, Yue Pang

**Affiliations:** 1 College of Life Science, Liaoning Normal University, Dalian, 116081 China; 2 Lamprey Research Center, Liaoning Normal University, Dalian, 116081 China; 3 Collaborative Innovation Center of Seafood Deep Processing, Dalian Polytechnic University, Dalian, 116023 China

**Keywords:** LIP, Transgenic zebrafish, Edema, Lipid peroxidation, Ferroptosis

## Abstract

**Background:**

Previously, a novel lamprey immune protein (LIP) was identified, which plays an important role in immunity and the regulation of growth and development in lampreys. However, the mechanism of how LIP regulates growth and development remains unclear.

**Methods:**

In this study, a zebrafish model of LIP overexpression was established by delivering a transgenic plasmid to the fertilized egg. The biological function of LIP was explored in vivo through phenotypic characterization, comparative transcriptome sequencing, and physiological and biochemical analyses.

**Results:**

LIP caused developmental toxicity in zebrafish, increased embryo mortality and exhibited strong teratogenic, lethal, and developmental inhibitory effects. Comparative transcriptome analysis showed that LIP-induced large-scale cell death by triggering ferroptosis. Furthermore, LIP-induced lipid peroxidation and caused pericardial edema. Direct inhibition of *acsl4a* and *tfr1a*, or silencing of *acsl4a* and *tfr1a* with specific siRNA suppressed ferroptosis and pericardial edema.

**Conclusions:**

Taken together, we confirmed that LIP can participate in growth and development via the regulation of lipid peroxidation and ferroptosis. This lays the foundation for future studies on the function of LIP in lampreys.

**Video Abstract**

**Supplementary Information:**

The online version contains supplementary material available at 10.1186/s12964-022-00933-0.

## Background

The origin of the adaptive immune system involves profound changes from invertebrates to vertebrates [1]. Lampreys belong to cyclostomes, are among the most primitive vertebrates, and can provide unique insights into adaptive immune system origins owing to their phylogenetic position at the base of the vertebrate tree [2]. In our previous studies, a novel lamprey immune protein (LIP) was identified as a highly expressed immune molecule from the supraneural body of lampreys, and the overall crystallographic structure of LIP was determined, showing an N-terminal lectin module and a C-terminal aerolysin module [3, 4]. The aerolysin-like protein Dln1, with similar structures, has been reported in zebrafish, suggesting a novel fish-specific defense molecule [5]. As a cytotoxic protein, LIP was found to have tumor-killing activity both in vitro and in vivo. Previous studies have shown that the cytotoxic action of LIP depends on phosphatidylserine content and reactive oxygen species (ROS) accumulation in the cell membrane [6]. In addition, LIP overexpression inhibits HeLa cell proliferation [6]. To date, most studies on LIP have only focused on cytotoxic function in vitro. Recently, we demonstrated that overexpression of miR-4561 can induce apoptosis of embryonic cells by targeting LIP, suggesting a role for LIP in embryonic development [7]. However, the mechanism by which LIP regulates growth and development remains unknown.

As a migratory creature, lampreys have a complex anadromous migration habit and life history, which greatly hinders the artificial breeding of lamprey. Larval lamprey lives in the substrate of freshwater streams for 2–12 years before metamorphosing into their juvenile parasitic form [8]. After metamorphosis, adults migrate into streams to reproduce in the spring [9], and they are highly selective over spawning habitats [10, 11]. Although *Lampetra* species successfully breed and develop into larvae, it is still very difficult for them to develop into adults [12, 13]. Hence, establishing a suitable lamprey model to understand the biological function of LIP is difficult.

Among all the conventional model animals, the evolutionary status of zebrafish is closest to that of lamprey. Basic organ patterning is conserved between lamprey and zebrafish; therefore, zebrafish are used to study the functional genes and organ systems of lamprey. Compared with lampreys, zebrafish have a stable genetic background, complete genome, and simple genetic operation. Furthermore, the rich mutagenesis library accumulated by previous genetic studies on zebrafish also provides conditions for studying functional genes [14]. In conclusion, the evidence suggests that constructing a transgenic zebrafish model is the best choice for studying the function of LIP.

In this study, a model of LIP-overexpressing transgenic zebrafish was established to explore the biological functions of LIP. The relationship between LIP and embryonic development is mediated by lipid peroxidation and ferroptosis. Therefore, Tg (TRE:EGFP-*lip*) can not only determine the role of LIP in the physiological and pathophysiological functions of ferroptosis in vivo, but also improve our understanding of the relationship between iron metabolism and lipid metabolism in lampreys. In addition, our work can lay the foundation for studying lipid metabolism and ferroptosis by LIP in lamprey and provide new insights into lamprey growth and development.

## Materials and methods

### Zebrafish husbandry and drug treatments

The AB strain (naive) of zebrafish was raised and maintained at 28.5 °C on a 14 h light/10 h dark cycle under standard laboratory conditions. Embryos were maintained at 28.5 °C in fish water (0.2% Instant Ocean Salt in deionized water). The embryos were washed and staged according to published guidelines [15].

Zebrafish at the blastula stage were considered to have developed normally and were selected for drug treatment for eight hours post-fertilization (hpf). Dox (Doxycycline Hyclate, Yeasen Biotech, China, 30 μg/mL) was used to induce the target gene. The treated embryos were protected from light, and the culture medium was changed daily.

### Generation of the transgenic zebrafish line

The pTRE-EGFP plasmid acquired from Clontech contained the second-generation rtTA- responsive element and minimal CMV promoter. The nucleotide sequence of MT176433.1 available in the NCBI database was used to synthesize *lip*. The pTol2-actb2-rtTAM2-TREP-EGFP-P2A-lip plasmid was constructed by the Nanjing Xinjia Medical Technology Co., Ltd. to construct. The PCR product and vector pTRE-EGFP were digested with KpnI and EcoRI and ligated using T4 DNA ligase to construct the pTRE-lip plasmid. A total of 50 pg plasmid was microinjected into each of the one-cell stage fertilized eggs. The injected embryos were incubated at 28.5 °C, and the hatched larvae were reared into adults. Total genomic DNA from adult fish tails was extracted and amplified using two pairs of PCR primers (Additional file 5: Table S1). The initial denaturation of DNA at 94 °C for 5 min was followed by 35 cycles of denaturation at 94 °C for 30 s, primer annealing at 55 °C for 30 s, and primer extension at 72 °C for 45 s. The final elongation step was performed at 72 °C for 10 min. PCR products were resolved using agarose gel electrophoresis.

### Phenotypic analysis

The experimental samples were separated into four groups. Naive and transgenic zebrafish embryos at the same embryonic stage, treated with 30 μg/mL Dox at 8 hpf, were used as the experimental groups; naive and transgenic zebrafish embryos without Dox treatment were used as the control groups. The naive control and experimental groups were used to determine the effect of Dox on the zebrafish phenotype, and the transgenic control and experimental groups were used to determine the effect of LIP overexpression on the zebrafish phenotype. All zebrafish embryos were cultured in the dark at 28.5 °C after collection.

Before performing morphological tests, zebrafish were rinsed thoroughly in fish water three times (5 min/wash) and anesthetized with 0.016% MS-222 (Tricaine methanesulfonate, Sigma-Aldrich, St. Louis, MO). Zebrafish were then oriented on their lateral side and mounted with 3% methylcellulose (C_20_H_38_O_11_, Meilunbio®, China) on a depression slide for observation using a stereomicroscope. The posture of the zebrafish was adjusted the eyes and body parts coincided as much as possible, and the tail and body were at the same level and suitable for observation. The situation was observed and recorded under a stereo microscope, and the zebrafish embryo survival rate, deformity rate, and deformity were counted. In addition, the body length, eyeball radius, and yolk sac area were recorded on 4 dpf and 7 dpf. The unhatched zebrafish larvae were stripped of the egg membrane before observation and recording.

The zebrafish heart was visible in front of the yolk sac and behind the jaw. The heart rate of normal zebrafish was identified by the heartbeat. Before measuring the heart rate of the zebrafish larva, it was placed at room temperature (28 °C) for 15 min to adapt to the ambient temperature. Then they were observed using a stereomicroscope and adjusted for the visibility of the heart. The number of zebrafish heartbeats was recorded every 20 s. The number of heartbeats was repeated three times to obtain the final value of the heartbeat per minute.

### Staining for cell death

Zebrafish embryos at 24, 48, 72 and 96 hpf were collected for the experiments. The embryos were immersed in 5 μg/mL PI (propidium iodide, Sigma) in fish water for 30 min. Next, the embryos were rinsed thoroughly in fish water three times (5 min/wash) and anesthetized with 0.016% MS-222 (Tricaine methanesulfonate, Sigma-Aldrich, St. Louis, MO). Afterward, they were oriented on their lateral side and mounted with 3% methylcellulose (C_20_H_38_O_11_, Meilunbio®, China) on a depression slide for observation using a zoom stereo microscope (SMZ1500, Nikon, Japan).

### RNA extraction, sequencing, and bioinformatics analysis

Total RNA from zebrafish embryos at 19, 36, 60, and 96 hpf (n = 15, each) was extracted using the mirVanaTM miRNA Isolation Kit (AM1561, AM1560, Ambion, USA) for transcriptome sequencing and qPCR. RNA extraction and sequencing (RNA-seq) were performed by the Shanghai OE Biotech Co., Ltd. Analyses of the RNA-seq data were performed following the standard procedures. The differentially expressed genes (DEGs) between samples were selected based on the following criteria: (1) the fold change was > 2, and (2) the false discovery rate (FDR) was < 0.05. To understand the functions of the DEGs, gene ontology (GO) functional enrichment and Kyoto Encyclopedia of Genes and Genomes (KEGG) pathway analysis were carried out using Goatools and KOBAS respectively.

### Quantitative real-time PCR (qPCR)

Quantitative PCR experiments were conducted using the SYBR® PrimeScript™ RT-PCR Kit (TaKaRa, China) according to the manufacturer’s protocol. Each reaction contained 1 × SYBR Premix Ex Taq, 10 μM of each primer, and 2 μL cDNA (50 ng/mL) in a final volume of 25 μL. Amplification was performed in a PCR Thermal Cycler Dice Real-Time System (TaKaRa, China) with the following parameters: initial denaturation at 95 °C for 30 s to activate the DNA polymerase, followed by 40 cycles of 5 s at 95 °C, 30 s at 60 °C, and 30 s at 72 °C. The gene primers and glyceraldehyde3-phosphate dehydrogenase (GAPDH)-specific primers are listed in Additional file 5: Table S1 (QPCR). GAPDH was used as an internal control. Each sample was analyzed in triplicate using the Thermal Cycler Dice Real-Time System analysis software (TaKaRa, China). The specificity of qPCR was validated using melting curve analysis.

### Western blot

Proteins were extracted from cells or tissues and subjected to 8% Criterion Precast Midi Protein Gel electrophoresis. Regular western procedures were used. Antibodies used for western blotting included anti-LIP rabbit polyclonal (laboratory preparation), anti-HMGB1 rabbit polyclonal (laboratory preparation), eGFP monoclonal antibody (F56-6A1.2.3, Invitrogen), goat anti-rabbit IgG (D111018), anti-ACTB mouse monoclonal antibody (D191047), anti-TP53 rabbit polyclonal antibody (D220082), anti-ACSL3 rabbit polyclonal antibody (D261226), and anti-ACSL4 rabbit polyclonal antibody (D121771). Unlabeled antibodies were purchased from Sangon Biotech Co., Ltd. (Shanghai, China).

### Metabolism analysis

There were two groups in the experiment: the control group consisted of naive zebrafish embryos, and the experimental group was transgenic zebrafish embryos. Tissue iron, MDA, GSH, and GSSG contents were measured using commercial assay kits (Beijing Solarbio Science & Technology Co., Ltd.). Zebrafish embryo and lamprey tissue samples (100 mg) were mechanically homogenized in 1 mL of the related lysis reagent for ferroptosis metabolic indicators. All the results were normalized to the corresponding total protein content.

### Regulation of ferroptosis in vivo

Vitamin E (Meilunbio®, China) was dissolved in a minimal amount of dimethyl sulfoxide (DMSO). Vitamin E (0.1 mM) and plain DMSO were used as the treatment and negative controls, respectively. The prepared reagent was dissolved in fish water for co-culture.

Ferric ammonium citrate (FAC, Meilunbio®, China) was dissolved in water. Deferoxamine mesylate (DFO, MedChemExpress), an iron chelator that binds free iron in a stable complex and prevents it from engaging in chemical reactions, was dissolved in a minimal amount of DMSO to a concentration of 10 mM (preservation solution). FAC (200 μg/mL) and DFO (100 μg/mL, 1:1785 dilution in PBS) were used as positive and treatment controls, respectively. The prepared reagent was dissolved in fish water for co-culture.

### siRNA interference and small molecule inhibition

siRNAs (1 nL, GenePharma Suzhou, China) were microinjected into one-cell stage zebrafish embryos. The siRNA sequences are listed in Additional file 5: Table S1. Rosiglitazone (10 μM ROSI, Meilunbio®, China), a classic peroxisome proliferator-activated receptor-γ agonist was used for ACSL4 inhibition [16]. Dihydroartemisinin (DHA, MedChemExpress), an inhibitor of *tfr* and also induces ferroptosis, was used to overexpress LIP in zebrafish [17].

### Image acquisition and statistical analysis

Embryos and larvae were analyzed using a fluorescence microscope (SMZ1500, Nikon, Japan) and subsequently photographed using digital cameras. Quantitative image analyses were performed using image-based morphometric analysis (NIS-Elements D3.1, Nikon, Japan) and ImageJ software (National Institutes of Health, Bethesda, MD, USA). Statistical analysis and graphical representation of the data were performed using GraphPad Prism 8.0 (GraphPad Software, San Diego, CA, USA). P values were calculated using ANOVA (GraphPad Prism) as specified.

## Results

### Identification and characterization of a LIP transgenic zebrafish model

**Fig. 1:**
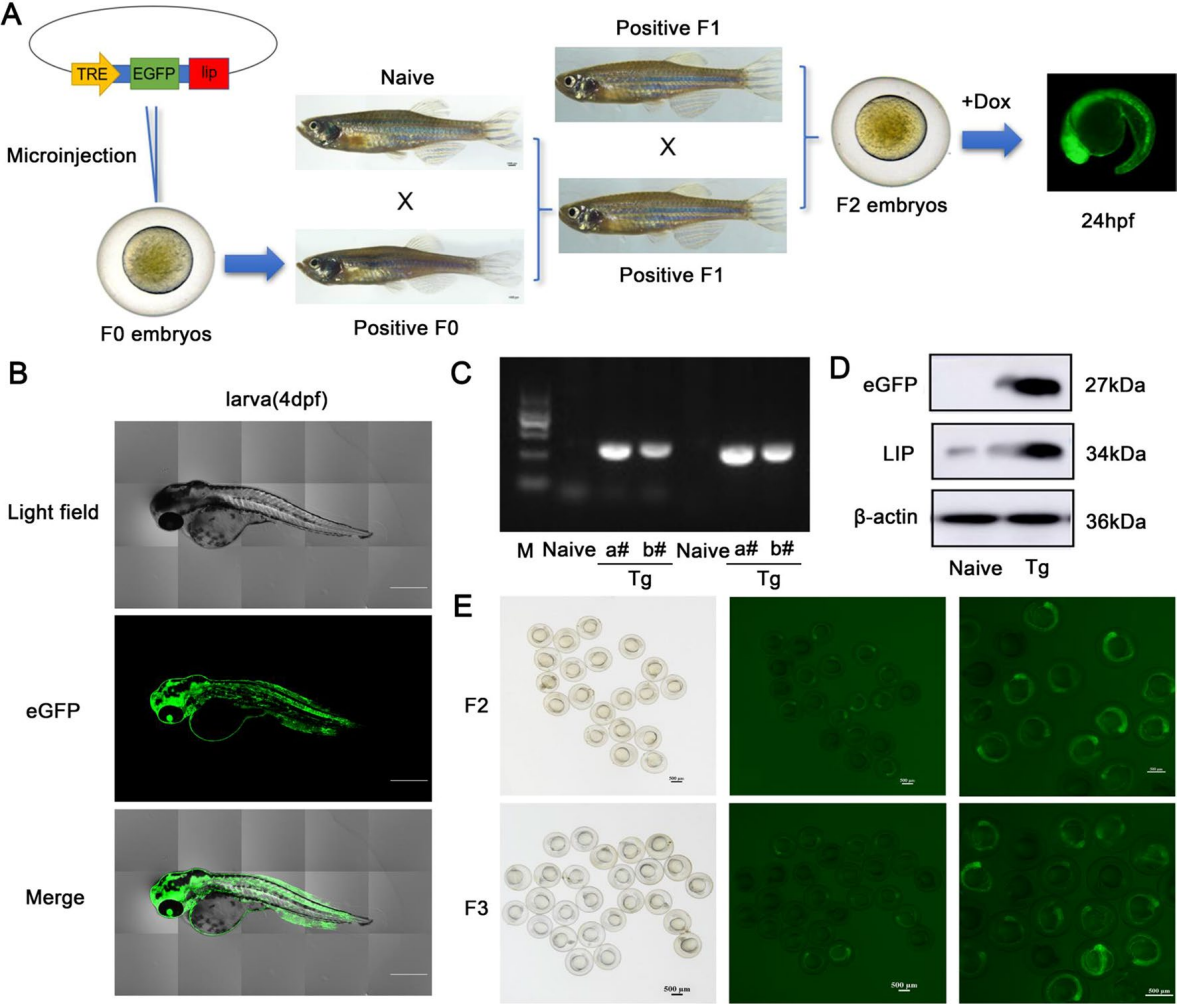
Establishment of a LIP-overexpression transgenic zebrafish model. **A** The plasmid was microinjected into fertilized eggs at one-cell stage. The positive F0 fish was individually crossed with naive fish to obtain positive F1 fish. F2 fish was reproduced by selfing the positive F1. Imaging EGFP expression in F2 embryos with or without Dox was performed using a fluorescence microscope. **B** Confocal imaging overexpression of fluorescent expression of LIP zebrafish. Scale 50 μm. **C** Positive F2 individuals were obtained by PCR screening. M: DNA ladder; lanes a# and b#: individual DNA samples (two transgenic lines with fluorescent expression obtained). The positive samples amplified 367–374 bp DNA bands. **D** Western blot analysis of LIP and EGFP in F2 fish. **E** Fluorescent expression efficiency of F2 and F3 generation transgenic zebrafish. Scale bar is 500 μm

To create a stable LIP transgenic zebrafish line with ubiquitous LIP expression, transgenic zebrafish were established using the Tet-On system for drug-induced overexpression of LIP (Fig. 1A). The transgenic zebrafish were obtained by injecting Tol2-actb2-rtTAM2-TREP-EGFP-P2A-*lip* plasmid into one-cell stage fertilized embryos. The transgenic zebrafish treated with Dox on 4dpf, high expression of green fluorescent protein (GFP) was observed (Fig. 1B). These larvae were reared into adulthood, and the genomic DNA from their tail fins was isolated for positive screening of *lip*. The positive rate of *lip* in F0 fish was 6.33% (Additional file 6: Table S2). These positive F0 fish were individually mated with naive fish to obtain their F1 offspring. F2 offsprings with high and stable fluorescence expression were obtained by self-crossing positive F1 fish. As shown in Fig. 1C, PCR detection indicated that *lip* was integrated into a single chromosomal locus. Western blot assay, demonstrated that transgenic zebrafish had both high levels of GFP and LIP expression (Fig. 1D), indicating that *lip* is stably inherited in the progeny of zebrafish. Fluorescence detection of different generations identified a stable line in the F4 generation (Fig. 1E, Additional file 6: Table S2).

### Overexpression of *lip* inhibits embryonic development of zebrafish

**Fig. 2:**
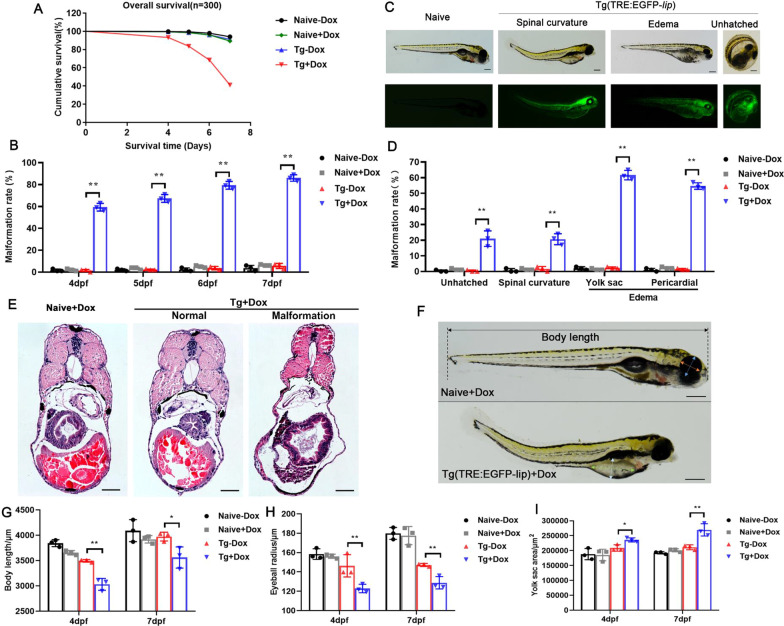
Overexpression of *lip* inhibits embryonic development of zebrafish. **A** The Keplan-meier survival curves of larvae (n = 100). P-values were obtained from two-sided log-rank tests. **B** The malformation rates at 4-7dpf (n = 100) of Dox-treated embryos were calculated and compared with those of their corresponding controls. **C** LIP overexpression causes 7dpf zebrafish to develop malformations. **D** The ratio of various malformations of 7dpf zebrafish (n = 100). **E** HE staining of zebrafish larvae with LIP overexpression malformation. **F** Lateral view of zebrafish with measurements of body length, eyeball diameter and yolk sac area. **G-I** Body lengths (**G**), eyeball diameters (**H**) and yolk sac area (**I**) of embryos injected with Tg(TRE:EGFP-*lip*) or naive control at 4 and 7dpf (n = 10). Data were given as means ± standard deviation. All figures are representative of three biological replicates. **, P < 0.01; *, 0.01 < P < 0.05. Scale bar is 250 μm

On 4-7dpf, Kaplan Meier survival curves for the LIP-overexpressing zebrafish group were significantly lower than those for the naive group (Fig. 2A). In addition, the malformation rate was significantly increased in LIP-overexpressing zebrafish than that in the naive zebrafish (Fig. 2B), indicating that LIP overexpression is lethal or malformation in zebrafish. In addition, no abnormal morphology or pericardial edema was observed in the naive (Fig. 2C), and severe morphological and developmental abnormalities were observed in transgenic zebrafish treated with Dox, such as spinal curvature, yolk sac edema, pericardium edema, and unhatched larvae (Fig. 2C, D). In the phenotypic analysis, edema was more significant than other malformations (Fig. 2D). In addition, H&E staining revealed that elevated pathology was also evident in LIP-overexpressing larvae in which edema and spinal curvature were increased (Fig. 2E). Furthermore, LIP overexpression caused significant growth retardation, including reductions in body length (Fig. 2F, G), eyeball radius (Fig. 2H), and yolk sac area (Fig. 2I). Collectively, LIP inhibits zebrafish development, including developmental retardation, and teratogenicity, and causes embryonic lethality.

### LIP overexpression causes massive cell death in the heart

**Fig. 3:**
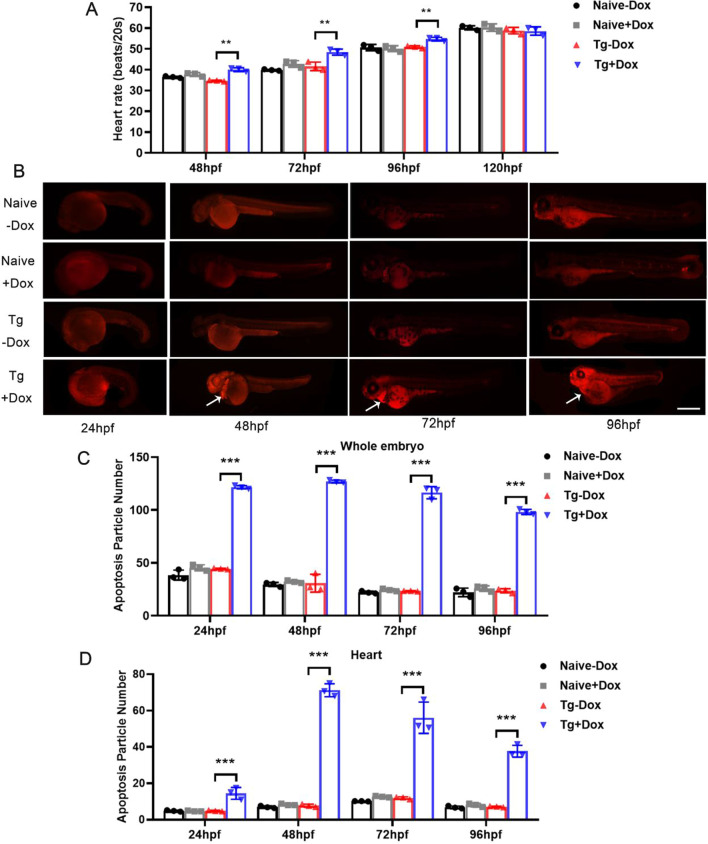
LIP inhibits embryonic development of transgenic zebrafish by inducing cell death. **A** Heart rate of 48-120hpf zebrafish every 20 s. **B** LIP overexpression induces effective cell death in whole embryos and heart. Zebrafish embryos were stained with propidium iodide (PI) at 24, 48, 72 and 96hpf. Death cells are visible as bright red spots, and less bright homogenous red staining, an unspecific background staining. Naive zebrafish exhibited few or no death cells in whole organism. In contrast, significantly increased staining was observed throughout the hearts in LIP overexpression embryos (white arrows). Scale bar is 500 μm. **C** Quantification of death particle number in whole embryo shows increase in LIP overexpression embryos at 24, 48, 72 and 96hpf. **D** Quantification of death particle number in heart shows increase in LIP overexpression embryos at 24, 48, 72 and 96hpf. Data were given as means ± standard deviation. All figures are representative of three biological replicates (per test n = 10). ***, P < 0.001; **, P < 0.01; *, 0.01 < P < 0.05

To determine the relationship of LIP with pericardial edema, heart rate was recorded in the whole process of Dox treatment. Our results demonstrated that heart rate fluctuations were not significantly different at 48 to 120 hpf in naive zebrafish. However, heart rate fluctuations in LIP-overexpressing transgenic zebrafish showed a significant increment at 48, 72, and 96 hpf (Fig. 3A). This indicates that LIP overexpression damages the heart function of zebrafish.

Next, cell death was detected using PI in zebrafish (Fig. 3B), and cell death particle numbers in transgenic zebrafish treated with Dox significantly increased in whole embryos compared to transgenic zebrafish treated without Dox (Fig. 3C). In addition, the number of cell death particles in the hearts of transgenic zebrafish with Dox treatment was significantly higher, especially at 48 hpf, and the heart was fully developed (Fig. 3D). These data suggest that LIP plays an inhibitory role in the growth and development of zebrafish via the control of heart function.

### Effect of LIP overexpression on zebrafish heart function by Ferroptosis pathway

**Fig. 4:**
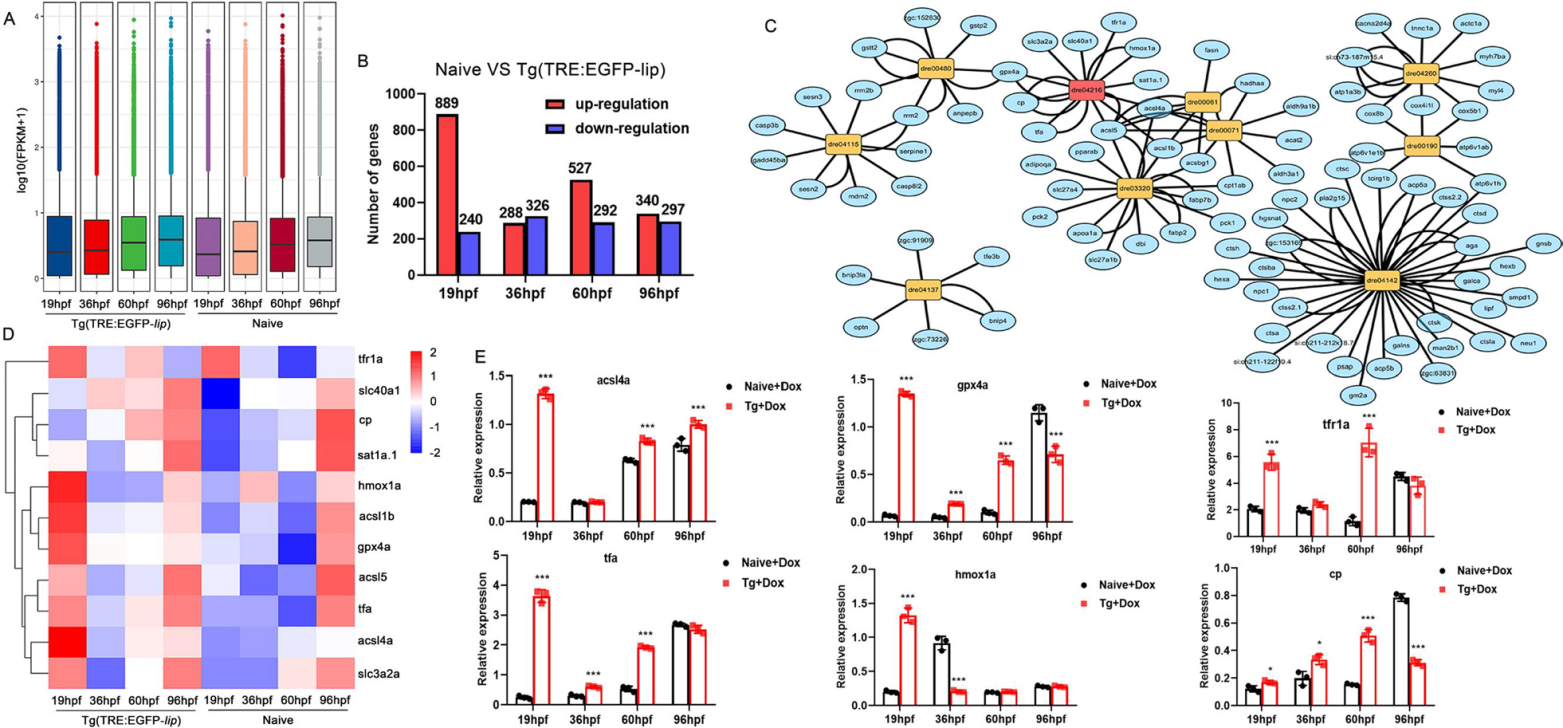
RNA-seq transcriptome analysis identifies a set of LIP-dependent targets in transgenic zebrafish. **A** Correlation coefficient of zebrafish at different stages of embryonic development. **B** The upregulated and downregulated DEGs in different periods. **C** KEGG pathway analysis of main DEGs in LIP overexpression groups, the red in the circle represents dominant influence DEG sets. **D** Heat map of RNA-seq showing genes related to ferroptosis pathway. **E** qPCR validation of differential expression of six genes. In E, data are presented as mean ± SEM, and all figures are representative of three biological replicates. ***, P < 0.001; *, 0.01 < P < 0.05

To further explore the role of LIP in embryonic development, we used transcriptome analysis, combined with functional validation, to reveal the potential biological functions of LIP from the perspective of functional genomics. Based on the embryonic development process, RNA-seq was performed with transgenic zebrafish at four relatively discrete stages, including myocardial progenitor cells forming a horseshoe-shaped structure (19 hpf, segmentation period), the heart begins to loop (36 hpf, hatching period), heart fully formed (60 hpf, hatching period), and complete most of its morphogenesis (96 hpf, early larvae). Transcriptome sequencing data are standardized after verification by fragments per kilobase per million (FPKM) (Fig. 4A). Following the screening standard, 1129, 614, 819, and 637 DEGs were obtained in comparisons between the naive and transgenic groups at different developmental stages, respectively. Among these DEGs, 889, 288, 527, and 340 DEGs were upregulated, and 240, 327, 292, and 297 DEGs were downregulated at 19, 36, 60, and 96 hpf, respectively (Fig. 4B). A total of 109 DEGs were common between the four groups of naive and transgenic zebrafish (Additional file 1: Fig. S1A). To reveal the dynamic change of LIP across different stages, we performed PCA (Additional file 1: Fig. S1B). We observed a continuous developmental process from *lip* expression in embryos (19 hpf) to the end of embryonic development (96 hpf). Differential gene expression analysis showed that different groups were enriched in functions that corresponded to features of LIP at different stages (Additional file 1: Fig. S1C).

Comparing the commonality of the four sets of transcriptomes, we found that LIP not only disturbs lipid metabolism through the PPAR signaling pathway, but also causes cell death via the ferroptosis and autophagy pathways. GO and KEGG pathway enrichment analyses suggested a potential impact of LIP protein overexpression on metabolic pathways involving lipid metabolism and cell death (Additional files 1 and 2: Figs. S1, S2). The ferroptosis pathway showed the strongest enrichment, followed by cogent signals that supported the KEGG analysis, including upregulation of ferroptosis marker molecules, such as *tf*, *tfr1a*, and *acsl4a*. The analysis of the molecular interaction network of ten major pathways differentially expressed in LIP transgenic zebrafish, which were divided into three groups: mitophagy, lysosome, and ferroptosis, demonstrated that these ten pathways were related to the ferroptosis pathway (Fig. 4C). Damage to mitochondria and lysosomes is a manifestation of ferroptosis, which is a form of cell death caused by lipid metabolism. Furthermore, molecules related to ferroptosis were significantly upregulated in the overexpressed LIP transcriptome (Fig. 4D). Therefore, we inferred that LIP triggered ferroptosis during embryonic development in zebrafish (Additional files 7 and 8: Tables S3, S4). The upregulation of ferroptosis-specific genes was confirmed by qPCR (Fig. 4E), which validated the reliability of our transcriptome-wide data.

### LIP overexpression in zebrafish larvae causes edema by triggering ferroptosis

**Fig. 5:**
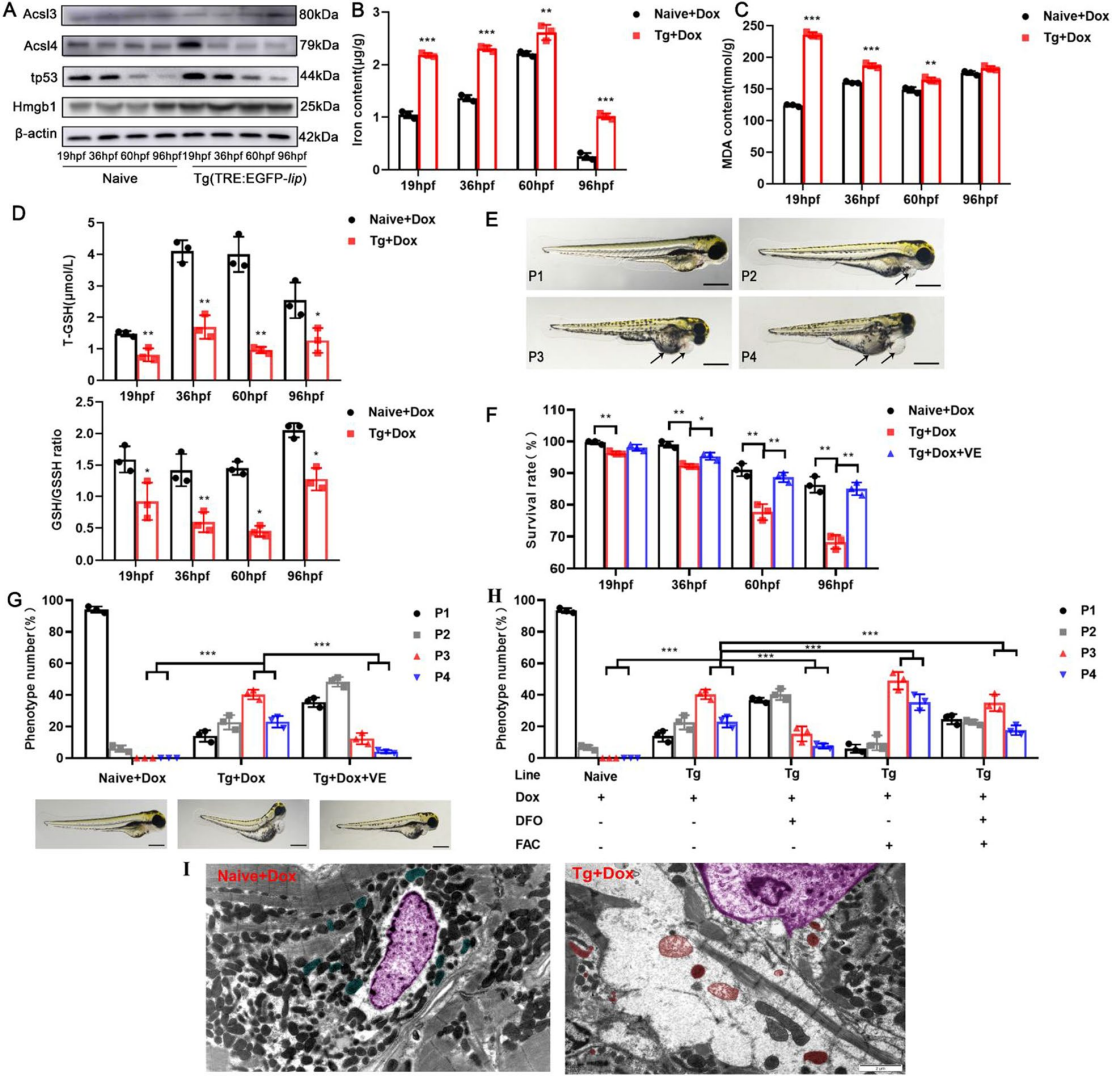
LIP overexpression in zebrafish larvae causes edema by triggering ferroptosis. **A** Ferroptosis expression in four stages of embryonic development of zebrafish was confirmed on Western Blot. **B-D** Zebrafish whole embryo iron (**B**), MDA (**C**), T-GSH and GSH/GSSG ratio (**D**) detection at 19, 36, 60 and 96hpf. **E** Representative phenotype of the larvae categorized into 4 groups: P1 = no edema, P2 = mild edema, P3 = severe edema, P4 = very severe edema. **F** Effects of vitamin E on survival rate of overexpressed LIP zebrafish. **G** Phenotypic classification and representative images of zebrafish larvae treated with vitamin at 96 hpf. **H** Phenotypic classification of 96hpf larvae treated by combination of FAC and DFO. **I** Transmission electron microscopy performed on heart sections from zebrafish myocardium demonstrates abnormal edema and mitochondria ultrastructure in LIP overexpression hearts. Pink: cell nucleus, blue: normal mitochondria, red: mitochondria after ferroptosis. Scale bar 2 μm. Data were given as means ± standard deviation. All figures are representative of three biological replicates (per test n = 100). ***, P < 0.001; **, P < 0.01; *, 0.01 < P < 0.05

We confirmed that LIP overexpression improved the expression of ACSL4, TP53, and HMGB1 at the protein level by immunoblotting (Fig. 5A). In addition, the contents of iron (Fig. 5B) and MDA (Fig. 5C) in overexpressed LIP zebrafish were significantly higher, and the ratios of GSH and GSH/GSSG (Fig. 5D) were significantly lower than those in the naive zebrafish. The results show that LIP triggers ferroptosis by modulating GSH metabolism, iron metabolism, and lipid peroxidation at multiple levels.

We then assessed the zebrafish phenotype, ranging from P1 = normal phenotype to P4 = very severe edema. More than 60% of LIP-overexpressing zebrafish developed a severe (P3 = 40.33%) or very severe edema phenotype (P4 = 23%, Fig. 5E). To investigate whether the edema phenotype was caused by ferroptosis, we used vitamin E as an inhibitor of ferroptosis in transgenic Tg (TRE:EGFP-*lip*) zebrafish that treat the edema phenotype. In vitamin E-treated zebrafish, a significant increase in survival and edema phenotypes were significantly lower than that in the untreated group, indicating that LIP overexpression in zebrafish larvae causes edema by triggering ferroptosis (Fig. 5F, G). To rule out the effect of the interaction between VE and LIP on the improved phenotype, we treated LIP-overexpressing zebrafish with FAC and DFO (a ferroptosis-inducing factor that induces cell death) and DFO (an iron-chelating agent that reduces the iron content in the body). Our results demonstrated that FAC caused edema in zebrafish, while DFO significantly inhibited the edema phenotype caused by FAC and LIP overexpression. Furthermore, we examined cardiomyocyte ultrastructure in vivo and found extensive mitochondrial damge and cell edema with obvious ferroptosis features (Fig. 5I). These results indicate that LIP overexpression in zebrafish larvae causes edema by triggering ferroptosis.

### Intervention of *tfr1a* and *acsl4a* to inhibit ferroptosis induced by LIP overexpression

TFR1 and ACSL4 are markers of ferroptosis and are upregulated in ferroptosis induced by LIP overexpression [18, 19]. Three siRNAs targeting tfr1a and acsl4a were designed to identify the direct target of LIP, qPCR using 24 hpf zebrafish embryos identified siRNA tfr1a-799 and siRNA acsl4a-275 as the ideal siRNA (Additional file 3: Fig. S3C–F) for follow-up experiments.

**Fig. 6:**
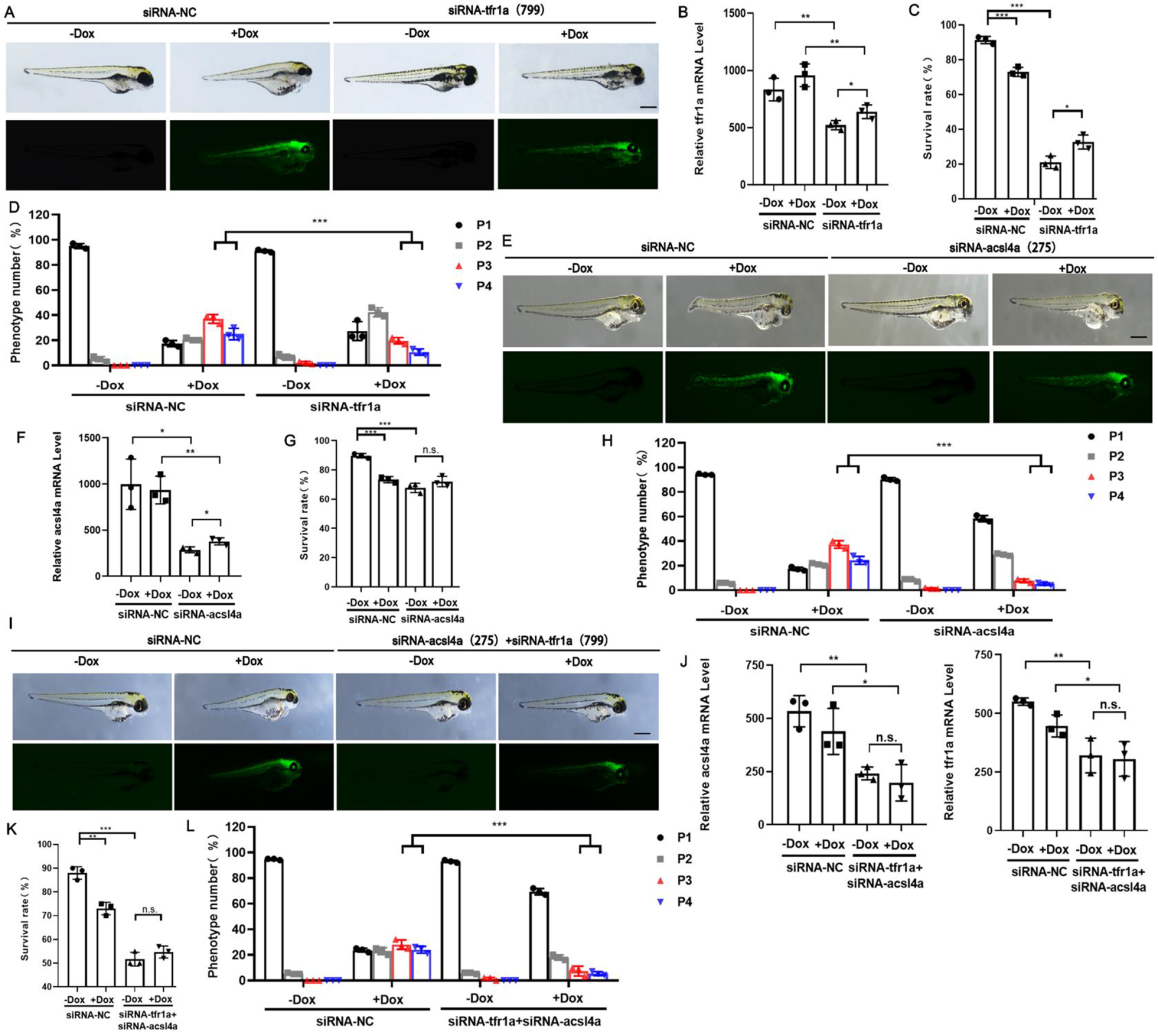
LIP overexpression induces ferroptosis through an increase in *tfr1a* and *acsl4a*. **A-D** SiRNA interference of zebrafish *tfr1a*. Representative phenotype (**A**), efficiency (**B**), survival rate (**C**) and phenotypic classification (**D**) of siRNA silenced zebrafish *tfr1a*. **E–H** SiRNA interference of zebrafish acsl4a. Representative phenotype (**E**), efficiency (**F**), survival rate (**G**) and phenotypic classification (**H**) of siRNA silenced zebrafish *acsl4a*. **I-L** SiRNA interference of zebrafish *tfr1a* and *acsl4a*. Representative phenotype (**I**), efficiency (**J**), survival rate (**K**) and phenotypic classification (**L**) of siRNA silenced zebrafish *tfr1a* and *acsl4a*. Data were given as means ± standard deviation. All figures are representative of three biological replicates (per test n = 100). ***, P < 0.001; **, P < 0.01; *, 0.01 < P < 0.05

As shown in Fig. 6A–E, knocking down two key ferroptosis genes, tfr1a and acsl4a, inhibited the edema phenotype of zebrafish larvae caused LIP-overexpression induced ferroptosis to a certain extent. Compared to siRNA tfr1a, siRNA acsl4a had a significant inhibitory effect (Fig. 6F–H), which is characterized by a high embryo survival rate, a significant decrease in severe edema phenotype, and a significant decrease in lip transcription level. Nevertheless, the mixed injection of the two siRNAs had a more significant inhibitory effect on the edema phenotype, indicating that LIP acts directly on acsl4a and indirectly on tfr1a (Fig. 6I–L). This suggests that LIP-overexpression induced ferroptosis can be inhibited by targeting tfr1a or acsl4a, and acsl4a may be the direct target of LIP.

**Fig. 7:**
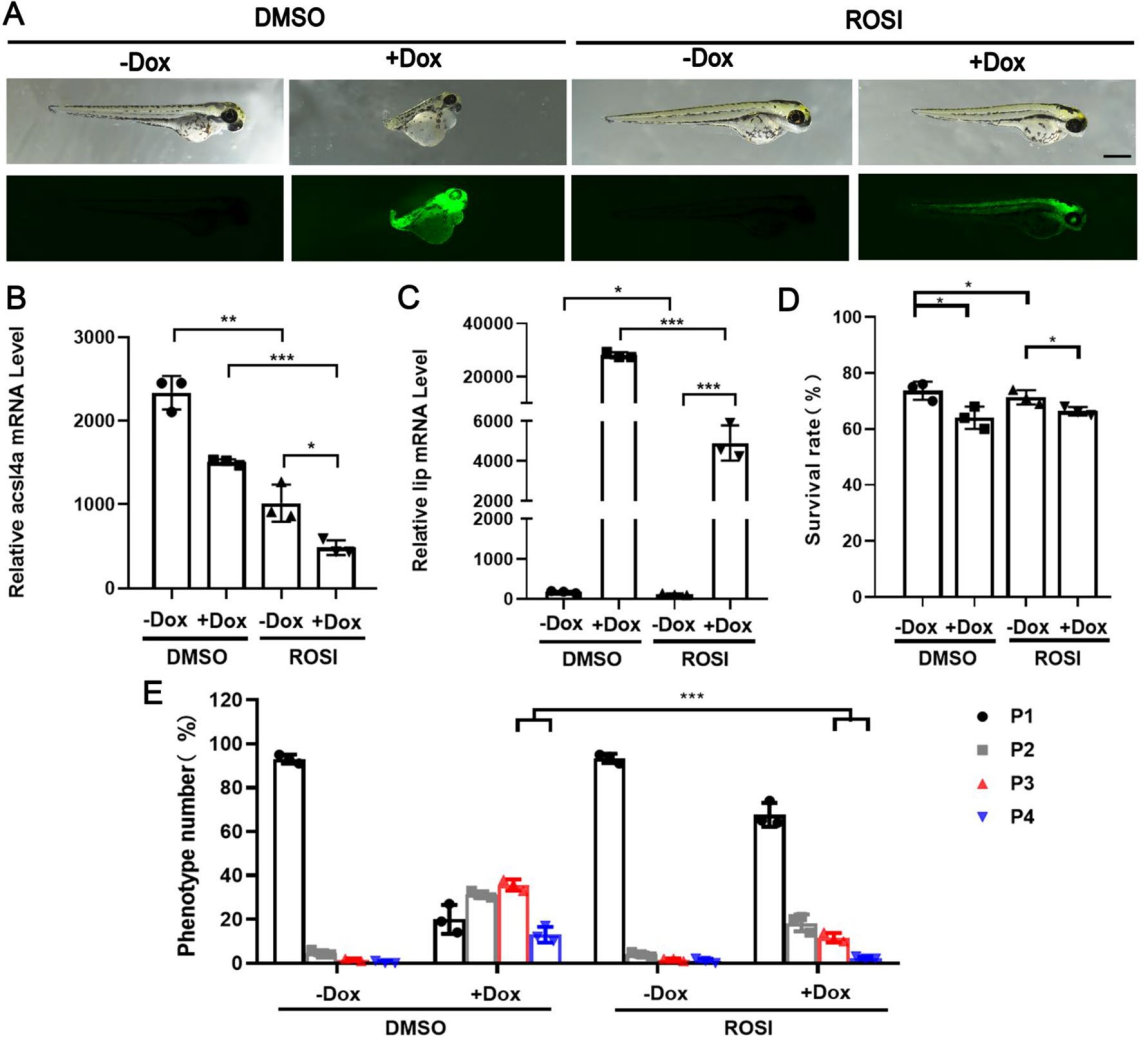
ROSI targeted *acsl4* inhibits ferroptosis. **A** Representative phenotypes of zebrafish after ROSI treatment. **B** Inhibition of ROSI on the transcription of zebrafish *acsl4a* gene. **C** Inhibition of ROSI on the transcription of zebrafish *lip* gene. **D** Survival rate of zebrafish after ROSI treatment. **E** Phenotypic classification of zebrafish after ROSI treatment. Data were given as means ± standard deviation. All figures are representative of three biological replicates (per test n = 100). ***, P < 0.001; **, P < 0.01; *, 0.01 < P < 0.05

To confirm the regulatory effect of ACSL4 on ferroptosis triggered by LIP, the LIP-overexpressing embryos were treated with ROSI, a small-molecule inhibitor of *acsl4a* [20]. Compared to the control group, the expression level of the *acsl4a* was significantly inhibited in the ROSI group (Fig. 7B). The qPCR results showed that the lip was also significantly downregulated and positively correlated with the expression levels of *acsl4a*. At the same time, inhibition of *acsl4a* resulted in the downregulation of *lip*, indicating that LIP acts directly on *acsl4a* (Fig. 7C). In addition, the abnormal edema phenotype of zebrafish embryos caused by LIP overexpression decreased significantly. The results showed that inhibiting the expression of *acsl4a* in zebrafish embryos had a protective effect on ferroptosis caused by overexpression of LIP, and could reduce the edema phenotype caused by overexpression of LIP (Fig. 7D, E). Similar experimental results were obtained using dihydroartemisinin (DHA) as an inducer of ferroptosis (Additional file 4: Fig. S4).

## Discussion

Our previous studies focused on the cytocidal activity of LIP against tumors. Additionally, LIP is involved in embryonic development through miR-4561 regulation; however, the mechanism by which LIP regulates development remains unclear. Here, we explored the mechanism of LIP regulation on the growth and development of zebrafish by constructing a LIP transgenic zebrafish model. Phenotypic and transcriptomic analyses of transgenic zebrafish Tg(TRE:EGFP-*lip*) demonstrate that LIP inhibits growth and development rather than participating only in immune defense. This study demonstrates a novel mechanism that LIP induces ferroptosis in other word, LIP regulates the development via its regulatory function in iron and lipid metabolism (Additional files 7 and 8: Tables S3, S4).

Inhibition of LIP inhibits development and causes embryonic lethality, abnormal morphogenesis of multiple tissues, and severe growth retardation, consequently adversely affecting the survival of zebrafish embryos. LIP triggers ferroptosis, which may result in the accumulation of tissue fluid or prostaglandins, eventually leading to edema [21, 22]. The resting heart rate is the most sensitive indicator of cardiac function [23]. LIP overexpression can greatly increase heart rate and cause cell death in the heart of zebrafish. The faster the heartbeat, the shorter the interval, the more susceptible the heart is to damage [24]. The occurrence of most congenital heart diseases is closely related to embryonic cell disorders [25]. Excessive ROS caused by oxidative stress is mostly produced in tissue cells with important functions, high activity, and high oxygen consumption, such as cardiac muscle cells, brain cells, and nerve cells. The higher free radical levels in these cells than that in other tissue cells lead to increased cell death in these cells [26]. It is speculated that LIP overexpression causes ROS explosion and oxidative stress damage, leading to cell death and pericardial edema.

The first evidence that LIP is involved in iron metabolism came from transcriptome studies of LIP overexpression in transgenic zebrafish. Pathways associated with the cardiovascular system, such as cardiac muscle contraction pathways, are downregulated. Simultaneously, pathways related to lipid metabolism, such as the PPAR pathway, are regulated [27]. Glucose and lipid metabolism disorders are the biggest threats to cardiovascular diseases [28]. This may be related to the inhibition of LIP on yolk granules and pericardial edema. Furthermore, LIP appears to influence the functions of other pathways through the ferroptosis pathway [29]. Ferroptosis is a new type of iron-dependent programmed cell death different from apoptosis, cell necrosis, and autophagy [30]. The main mechanism of ferroptosis is that under the action of divalent iron or ester oxygenase, the unsaturated fatty acids with a high expression on the cell membrane are catalyzed to induce lipid peroxidation, thus inducing cell death [31]. In addition, it is also manifested in the reduction of the core enzyme GPX4 in the regulation of the antioxidant system (glutathione system) [32]. Previous studies have shown that ACSL4 leads to an outbreak of lipid peroxidation through the PPAR pathway [33]. TFR1 promotes iron overload via the ferroptosis pathway [34]. Our study demonstrated that LIP is involved in the regulation of acsl4a and tfr1a, thus regulating ferroptosis. P53 regulates ferroptosis through transcriptional or posttranslational mechanisms [35]. This may be related to the tumor-killing activity of the LIP. Thus, it is speculated that LIP plays an important role in immune defense and inhibits growth and development by regulating lipid metabolism and iron metabolism. The occurrence of lipid peroxidation can be determined by detecting MDA, a marker of lipid peroxidation [36, 37]. In this study, the increased MDA content indicated high levels of iron and lipid peroxidation in LIP-overexpressing transgenic zebrafish.

The inherent characteristics of ferroptosis suggest that it regulates tissue homeostasis and growth. Studies have demonstrated that ferroptosis is triggered by degenerative processes or may be induced therapeutically in some cancers, but few studies have explored its natural functions [38]. Compared with other fish, lampreys feed on iron-rich blood [39]. Consequently, lampreys are more prone to age-related iron accumulation [40]. To counter this risk, LIP may trigger ferroptosis to maintain the iron balance in the body. It is well known that the liver is a major iron reservoir, and the liver of lampreys is rich in iron and fat, suggesting that lampreys require special mechanisms to inhibit hepatofibrosis and fatty liver [41]. Zhang et al. found that ferroptosis in HSCs can effectively inhibit hepatofibrosis [42]. Ferroptosis involves lipid and iron metabolism, which are closely related to growth and development. Overexpression of LIP mobilizes a large amount of stored iron, resulting in increased blood iron concentration, growth inhibition, and ferroptosis. The consumption of iron may cause iron deficiency, which causes nutritional anemia and may also contribute to poor development [43]. In addition, ferroptosis leads to myocarditis, which is associated with the pericardial edema phenotype in zebrafish [44]. Simultaneously, the iron imbalance can lead to abnormal bone metabolism, which is related to the spine curvature phenotype of zebrafish [45]. Clarification of the definitive role of LIP in ferroptosis will provide insights into of the origin and dual role of LIP in immune defense and growth. Although research has revealed that LIP is related to immune recognition and defense, the role of LIP in the growth and development of lampreys remains unclear. It is of great significance to clarify the specific role of LIP in triggering ferroptosis, as it helps to explain the dual role of LIP in immune defense and growth.

## Conclusions

**Fig. 8:**
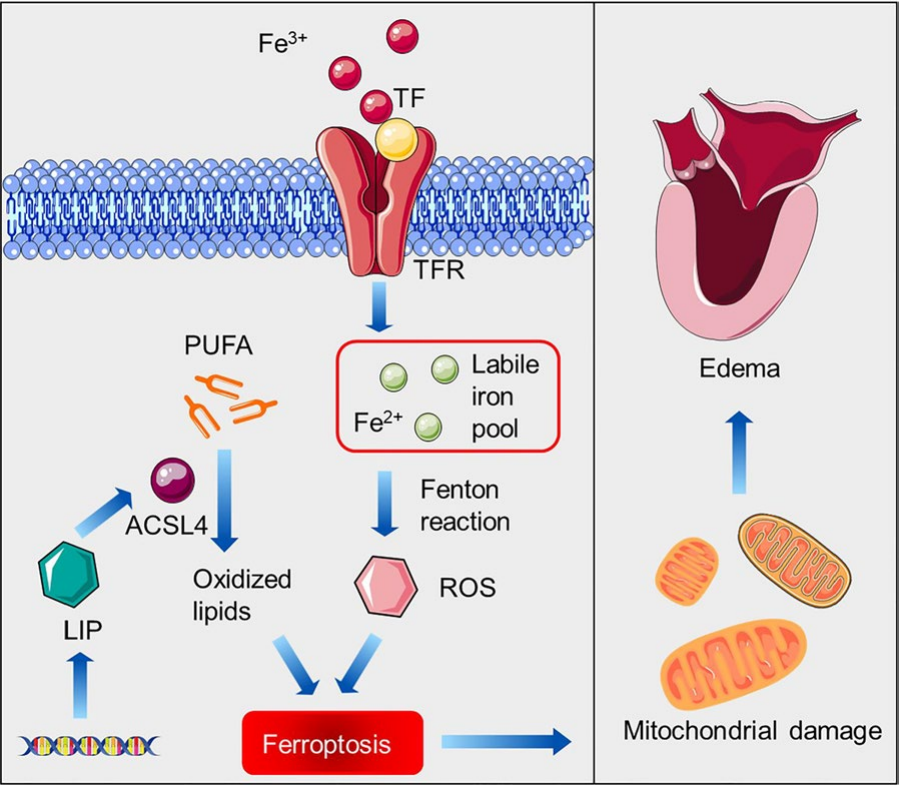
Network visualization of key signaling and *lip* regulatory modules implicated in ferroptosis. The gene regulatory network comprises the following circuits (in chronological order of execution): 1. Iron overload; 2. Lipid peroxidation 3. Ferroptosis and 4. Mitochondrial damage and pericardial edema. Full protein names of the nodes in this network are as follows: TF: transferrin, TFR: transferrin receptor, ACSL4: acyl-CoA synthetase long chain family member 4

In summary, our data constructed a transcriptome of LIP transgenic zebrafish and demonstrated that LIP inhibits embryo development by triggering ferroptosis, providing an animal model for studying LIP and ferroptosis. Importantly, we elucidated that LIP directly targets *acsl4* and mediates lipid peroxidation to trigger ferroptosis. Considerable progress has been made regarding the biological functions of LIP in lampreys. A schematic illustration of LIP functions is shown in Fig. 8. Further elucidation of the exact molecular mechanisms of ferroptosis and developmental delay caused by LIP will help us understand the basic processes of iron metabolism, which may contribute to developing new treatments for hemochromatosis and developmental and neurodegenerative diseases.

## Supplementary Information

**Additional file 1. Fig. S1**: RNA-seq transcriptome analysis identifies a set of LIP-dependent targets in transgenic zebrafish. (A) Venn diagram of DEGs between four zebrafish embryonic developmental stages. (B) Three-dimensional (3D) PCA of lip overexpression at distinct stages shows the progressive expression of lip. (C) Heat map showing the expression of different stage-specific genes (FDR < 0.001) and gene ontology (GO) analysis showing the functional enrichment of lip. (D) As the dominant KEGG pathway, the ferroptosis pathway shows relevant DEGs of fold change. (E-H) Enrichment of GO terms among LIP-interactors identified in LIP overexpression zebrafish. The associated GO term, enrichment score, and the number of contributing genes for the main enriched clusters. The red box indicates the main enrichment clusters. Source data are available online for this figure

**Additional file 2. Fig. S2**: Enrichment of KEGG terms among LIP-interactors identified in LIP overexpression zebrafish. (A-D) Shown are the associated KEGG term, enrichment score, and number of contributing genes for the main enriched clusters. The red box indicates the main enrichment clusters. (E-H) Shown are the associated KEGG term, enrichment score, and number of contributing genes for the top 20-enriched clusters. The red box indicates the main enrichment clusters. Source data are available online for this figure

**Additional file 3. Fig. S3**: The mechanism of ferroptosis in LIP overexpression zebrafish. (A) Western blot quantitative analysis of ferroptosis proteins in zebrafish during embryonic development. (B-D) Validation of VE, FAC and DFO in the regulation of zebrafish ferroptosis. Iron content(B), mRNA (C) and expression (D) levels of ferroptosis marker molecule acsl4 detected. (E) Survival rate of three siRNAs interfered with zebrafish acsl4a expression. (F) The expression efficiency of three siRNAs interfered with the expression of zebrafish acsl4a. (G) The expression efficiency of three siRNAs interfered with the expression of zebrafish tfr1a. (H) Survival rate of three siRNAs interfered with zebrafish tfr1a expression. Data were given as means ± standard deviation. All figures are representative of three biological replicates (per test n=10). ***, P<0.001; **, P<0.01; *, 0.01<P<0.05. Source data are available online for this figure

**Additional file 4. Fig. S4**: DHA targeted tfr induces ferroptosis. (A) Representative phenotypes of zebrafish after DHA treatment. (B) Inhibition of DHA on the transcription of zebrafish tfr1a. (C) Inhibition of DHA on the transcription of zebrafish lip. (D) Survival rate of zebrafish after DHA treatment. (E) Phenotypic classification of zebrafish after DHA treatment. Data were given as means ± standard deviation. All figures are representative of three biological replicates (per test n=10). ***, P<0.001; **, P<0.01; *, 0.01<P<0.05. Source data are available online for this figure

**Additional file 5. Table S1**: Primer information of *Danio rerio*

**Additional file 6. Table S2**: Germline transmission and frequency of *lip* gene

**Additional file 7. Table S3**: Summary of sequence data generated for zebrafish transcriptome and quality filtering

**Additional file 8. Table S4**: Top 10 differentially up or down-regulated KEGG pathways in four stages of embryonic development

## Data Availability

All data generated or analysed during this study are included in this published article [and its Additional files]. All relevant data are available from the corresponding author upon reasonable request. The datasets presented in this study can be found in online repositories. The names of the repository/repositories and accession number(s) can be found below: NCBI, PRJNA782739.

## Abbreviations

LIP: Lamprey immune protein
ROS: Reactive oxygen species
miR-4561: MicroRNA-4561
Dox: Doxycycline Hyclate
hpf/dpf: Hour/day post-fertilization
MS-222: Tricaine methanesulfonate
FDR: False discovery rate
GAPDH: Glyceraldehyde-3-phosphate dehydrogenase
HMGB1: High mobility group box-1 protein
eGFP: Enhanced green fluorescent protein
ACTB: β-Actin
TP53: Tumor Protein P53
ACSL3: Acyl-CoA Synthetase Long Chain Family Member 3
ACSL4: Acyl-CoA Synthetase Long Chain Family Member 4
MDA: Malondialdehyde
GSH: Glutathione
GSSG: L-Glutathione Oxidized
DMSO: Dimethyl sulfoxide
FAC: Ammonium ferric citrate
DFO: Deferoxamine mesylate
ROSI: Rosiglitazone
DHA: Dihydroartemisinin
FPKM: Fragments per kilobase per million
DEGs: Differentially expressed genes
TFR1: Transferrin receptor 1
GPX4: Glutathione peroxidase 4
