# Supplementary figures and images for: Lamprey immune protein triggers the ferroptosis pathway during zebrafish embryonic development
Source: Cell Commun Signal. 2022 Aug 17;20:124. doi: 10.1186/s12964-022-00933-0 (PMC9386916; doi:10.1186/s12964-022-00933-0)

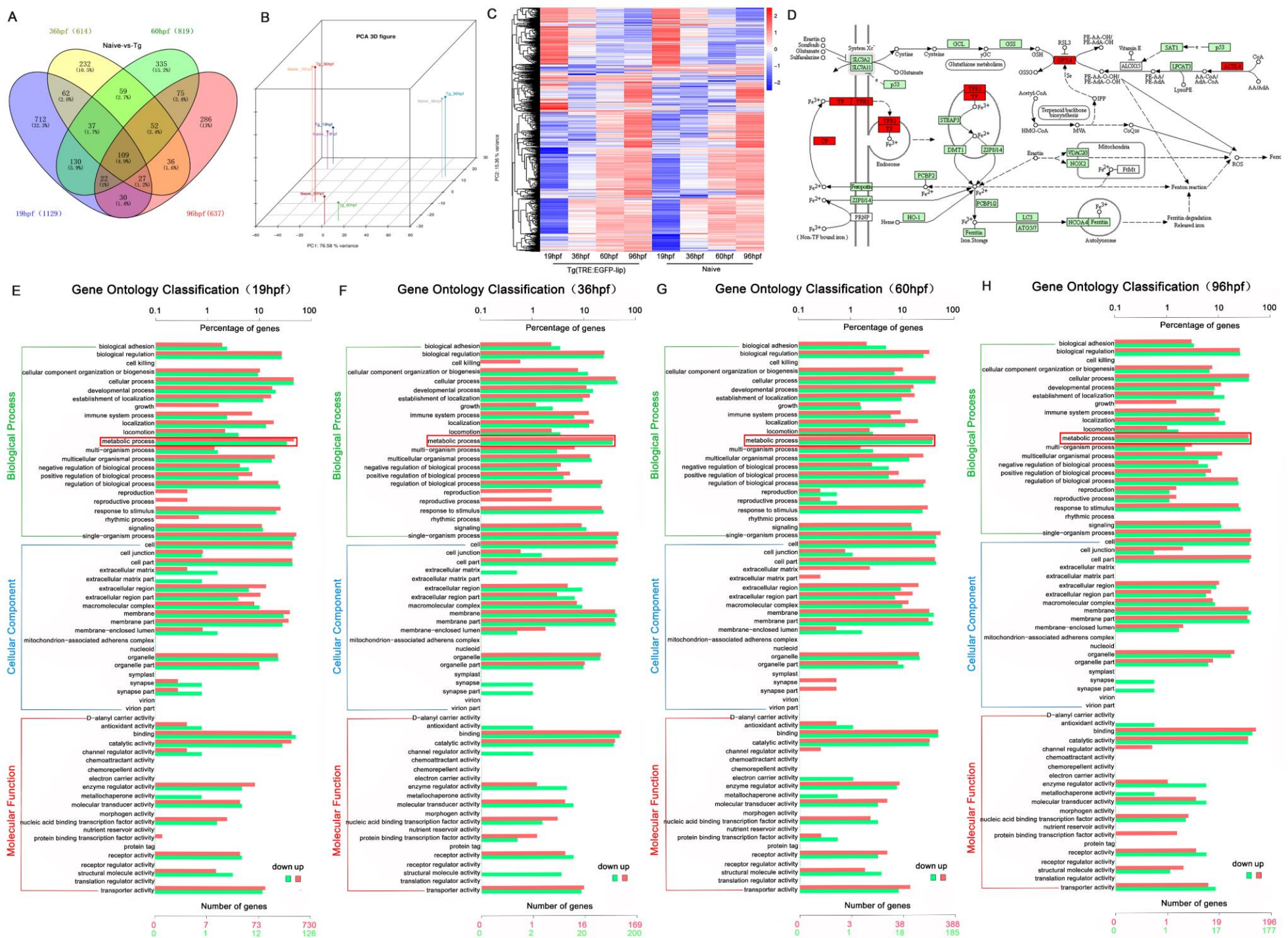

Supplement: Supplementary file 2 — Additional file 1. Fig. S1: RNA-seq transcriptome analysis identifies a set of LIP-dependent targets in transgenic zebrafish. (A) Venn diagram of DEGs between four zebrafish embryonic developmental stages. (B) Three-dimensional (3D) PCA of lip overexpression at distinct stages shows the progressive expression of lip. (C) Heat map showing the expression of different stage-specific genes (FDR < 0.001) and gene ontology (GO) analysis showing the functional enrichment of lip. (D) As the dominant KEGG pathway, the ferroptosis pathway shows relevant DEGs of fold change. (E-H) Enrichment of GO terms among LIP-interactors identified in LIP overexpression zebrafish. The associated GO term, enrichment score, and the number of contributing genes for the main enriched clusters. The red box indicates the main enrichment clusters. Source data are available online for this figure [file 12964_2022_933_MOESM2_ESM.pdf]

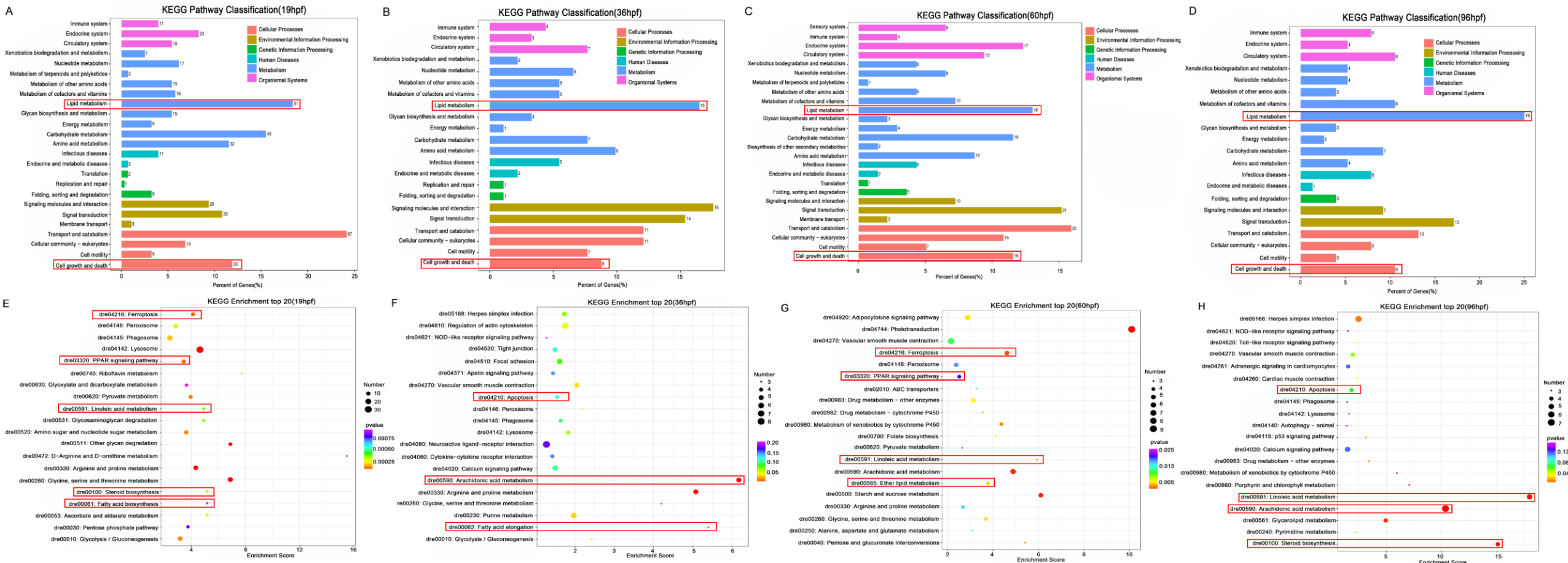

Supplement: Supplementary file 3 — Additional file 2. Fig. S2: Enrichment of KEGG terms among LIP-interactors identified in LIP overexpression zebrafish. (A-D) Shown are the associated KEGG term, enrichment score, and number of contributing genes for the main enriched clusters. The red box indicates the main enrichment clusters. (E-H) Shown are the associated KEGG term, enrichment score, and number of contributing genes for the top 20-enriched clusters. The red box indicates the main enrichment clusters. Source data are available online for this figure [file 12964_2022_933_MOESM3_ESM.pdf]

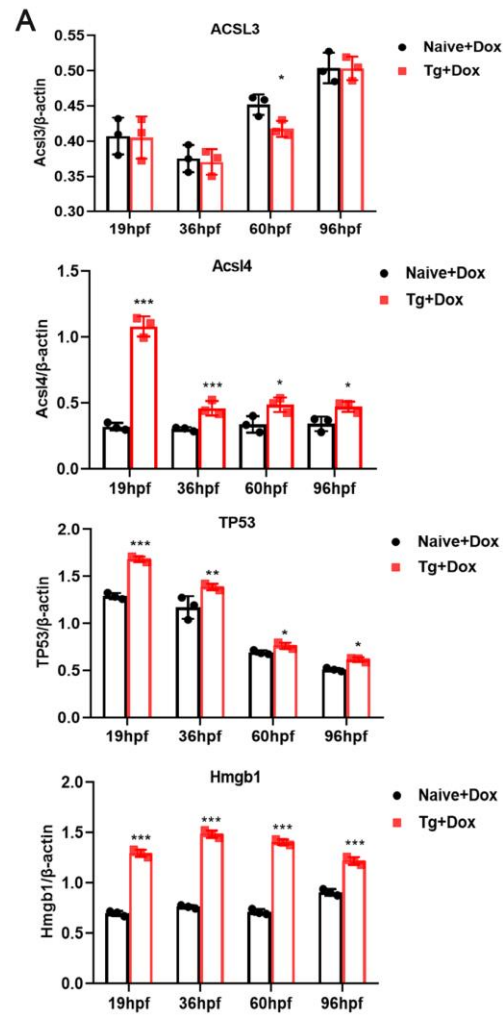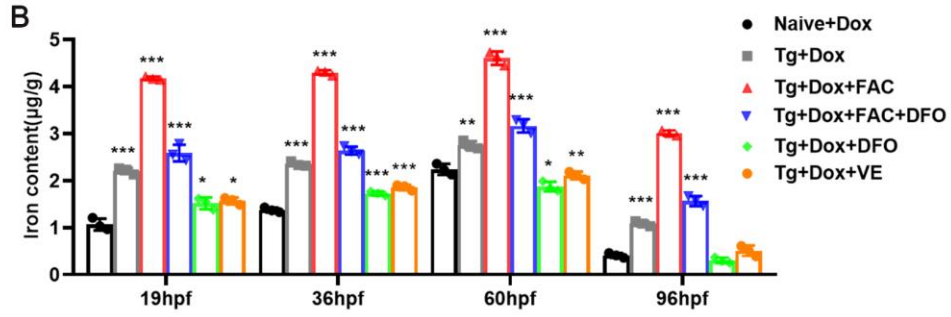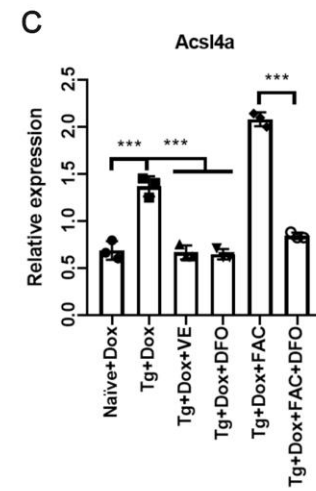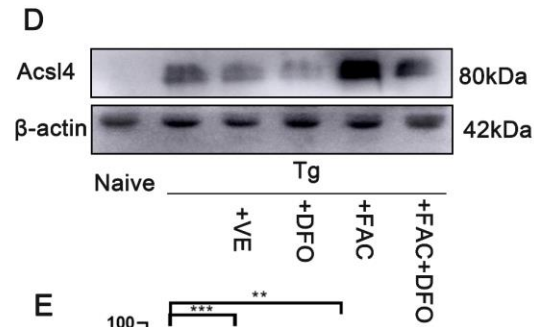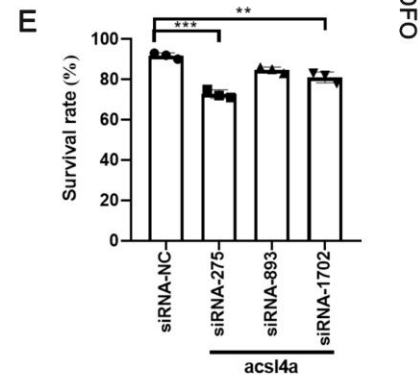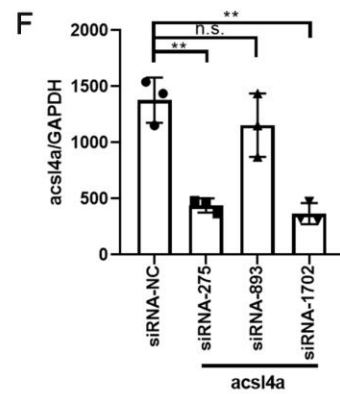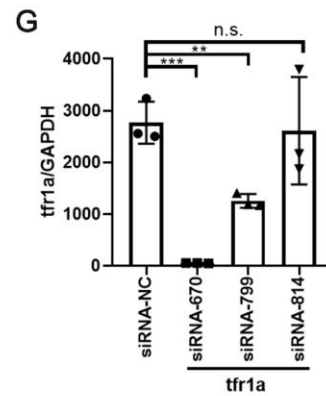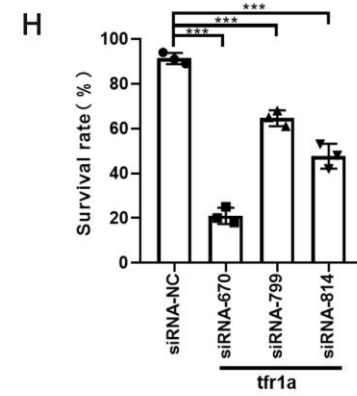

Supplement: Supplementary file 4 — Additional file 3. Fig. S3: The mechanism of ferroptosis in LIP overexpression zebrafish. (A) Western blot quantitative analysis of ferroptosis proteins in zebrafish during embryonic development. (B-D) Validation of VE, FAC and DFO in the regulation of zebrafish ferroptosis. Iron content(B), mRNA (C) and expression (D) levels of ferroptosis marker molecule acsl4 detected. (E) Survival rate of three siRNAs interfered with zebrafish acsl4a expression. (F) The expression efficiency of three siRNAs interfered with the expression of zebrafish acsl4a. (G) The expression efficiency of three siRNAs interfered with the expression of zebrafish tfr1a. (H) Survival rate of three siRNAs interfered with zebrafish tfr1a expression. Data were given as means ± standard deviation. All figures are representative of three biological replicates (per test n=10). ***, P<0.001; **, P<0.01; *, 0.01<P<0.05. Source data are available online for this figure [file 12964_2022_933_MOESM4_ESM.pdf]

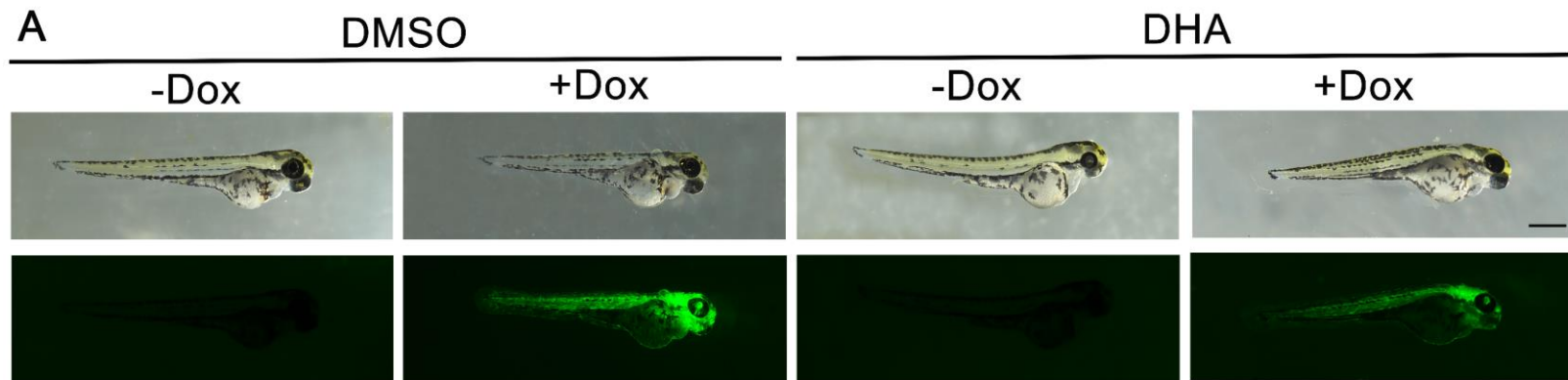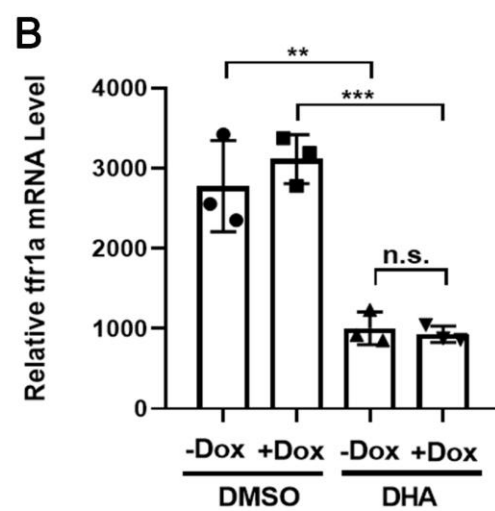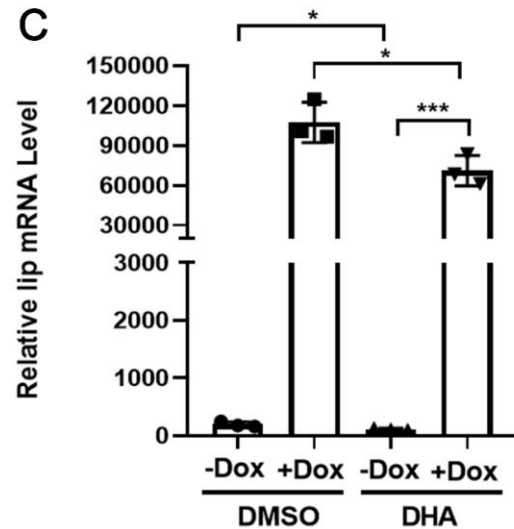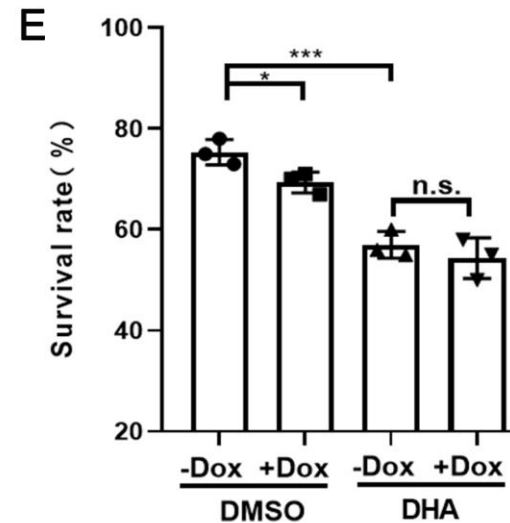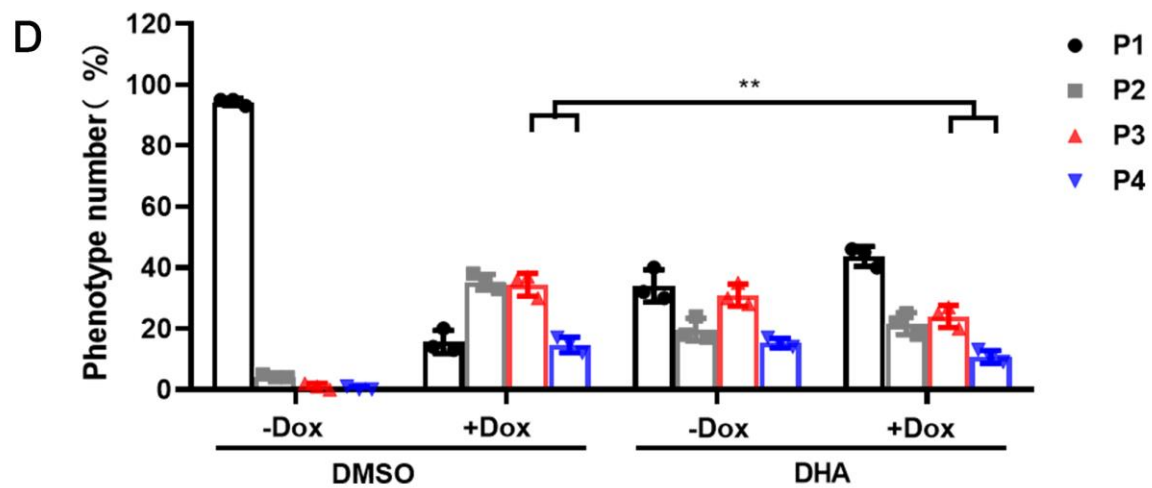

Supplement: Supplementary file 5 — Additional file 4. Fig. S4: DHA targeted tfr induces ferroptosis. (A) Representative phenotypes of zebrafish after DHA treatment. (B) Inhibition of DHA on the transcription of zebrafish tfr1a. (C) Inhibition of DHA on the transcription of zebrafish lip. (D) Survival rate of zebrafish after DHA treatment. (E) Phenotypic classification of zebrafish after DHA treatment. Data were given as means ± standard deviation. All figures are representative of three biological replicates (per test n=10). ***, P<0.001; **, P<0.01; *, 0.01<P<0.05. Source data are available online for this figure [file 12964_2022_933_MOESM5_ESM.pdf]
