# Supplementary material for: Lamprey immune protein triggers the ferroptosis pathway during zebrafish embryonic development
Source: Cell Commun Signal. 2022 Aug 17;20:124. doi: 10.1186/s12964-022-00933-0 (PMC9386916; doi:10.1186/s12964-022-00933-0)
Supplement: Supplementary file 6 — Additional file 5. Table S1: Primer information of Danio rerio [file 12964_2022_933_MOESM6_ESM.pdf]

Table S1 Primer information of *Danio rerio*.

Primer Name	Forward primer (5’/3’)	Reverse primer (5’/3’)	Serial number	Use
hmox1a	GAAACGCAGACCACCCAGCAG	CGGCAGGCACTGTTATTCTCTTCC	NM_001127516.1	Real-time PCR
gpx4a	TGCGACACCACAACTGACTGAAC	ACGTTGCACCAATGATCCCACTG	NM_001346537.1	
acsl4a	AGCGGACTGGCAGCACTAGG	GAAGCAGGCTTGAGCGGTGATC	NM_200649.1	
tfa	TCTTTGCTGGGATGTCTGGTTGTG	TGCTGCTTTGGTGGCGAGATG	NM_001291499.1	
cp	AGGTCCCGTCATTAGAGCAGAGG	CTCCGTGTGGCTGAATACTGAAGG	NM_131802.1	
gapdh	AACCAACTGCCTGGCTCCT	GTCTTCTGCGTTGCCGTG	KU041137.1	
lip-1	TGAAGTACCCGACCATGCAC	CGTGTACGGAAGGTCGATGT	AFX60113.1	PCR
lip-2	GCATCGTGCAATCGTACACC	GTGAGCCCCTTGTAGATGCC		
Negative control	UUCUCCGAACGUGUCACGUTT	ACGUGACACGUUCGGAGAATT		siRNA
tfr1a- 670	GCAGCUCUGUAUGCCCAUUTT	AAUGGGCAUACAGAGCUGCTT	XM_005163666.4	
tfr1a- 799	GCUGCUAGCUUGAAUGCUATT	UAGCAUUCAAGCUAGCAGCTT		
tfr1a-814	GCUAGUGCCGUGCUGAUUUTT	AAAUCAGCACGGCACUAGCTT		
acsl4a-275	GCUUUCUGCCAUGGUACUUTT	AAGUACCAUGGCAGAAAGCTT	NM_200649.1	
acsl4a- 893	GCAUGAACACAGCAGGAUUTT	AAUCCUGCUGUGUUCAUGCTT		
acsl4a-1702	GCAGAAGGUGGUUAUACAATT	UUGUAUAACCACCUUCUGCTT		
