# Supplementary material for: Lamprey immune protein triggers the ferroptosis pathway during zebrafish embryonic development
Source: Cell Commun Signal. 2022 Aug 17;20:124. doi: 10.1186/s12964-022-00933-0 (PMC9386916; doi:10.1186/s12964-022-00933-0)
Supplement: Supplementary file 7 — Additional file 6. Table S2: Germline transmission and frequency of lip gene [file 12964_2022_933_MOESM7_ESM.pdf]

Table S2 Germline transmission and frequency of *lip* gene.

Generation	Positive fish number	Total fish number	Positive frequency（%）
F0	19	300	6.33
F1	36	258	13.95
F2	121	216	56.02
F3	152	169	89.94
F4	176	176	100
