# Supplementary material for: Lamprey immune protein triggers the ferroptosis pathway during zebrafish embryonic development
Source: Cell Commun Signal. 2022 Aug 17;20:124. doi: 10.1186/s12964-022-00933-0 (PMC9386916; doi:10.1186/s12964-022-00933-0)
Supplement: Supplementary file 8 — Additional file 7. Table S3: Summary of sequence data generated for zebrafish transcriptome and quality filtering [file 12964_2022_933_MOESM8_ESM.pdf]

Table S3 Summary of sequence data generated for zebrafish transcriptome and quality filtering.

Sample	Clean reads	Total mapped reads	Uniquely mapped rate (%)	Q30 percentage (%)	GC percentage (%
Naive_19hpf	54.94M	50.68M	89.05%	92.20%	46.58%
Naive_36hpf	51.18M	47.31M	87.54%	92.50%	46.61%
Naive_60hpf	56.27M	51.93M	87.39%	92.24%	46.35%
Naive_96hpf	49.34M	45.31M	87.61%	92.27%	46.32%
Tg_19hpf	50.99M	47.34M	89.42%	92.32%	46.67%
Tg_36hpf	50.32M	46.69M	87.62%	92.04%	46.45%
Tg_60hpf	53.59M	49.65M	87.56%	92.26%	46.17%
Tg_96hpf	51.32M	47.38M	88.35%	92.20%	46.29%
