# Supplementary material for: Lamprey immune protein triggers the ferroptosis pathway during zebrafish embryonic development
Source: Cell Commun Signal. 2022 Aug 17;20:124. doi: 10.1186/s12964-022-00933-0 (PMC9386916; doi:10.1186/s12964-022-00933-0)
Supplement: Supplementary file 9 — Additional file 8. Table S4: Top 10 differentially up or down-regulated KEGG pathways in four stages of embryonic development [file 12964_2022_933_MOESM9_ESM.pdf]

Table S4 Top 10 differentially up or down-regulated KEGG pathways in four stages of embryonic development.

	Pathways	Number of DEGs	P-Value	Pathway ID
19hpf	Lysosome	31	3.25E-14	dre04142
	Glycine, serine and threonine metabolism	15	1.05E-10	dre00260
	Other glycan degradation	8	8.72E-07	dre00511
	Arginine and proline metabolism	13	9.80E-07	dre00330
	D-Arginine and D-ornithine metabolism	3	5.35E-06	dre00472
	Ferroptosis	9	4.19E-05	dre04216
	PPAR signaling pathway	11	6.05E-05	dre03320
	Pyruvate metabolism	9	6.24E-05	dre00620
	Amino sugar and nucleotide sugar metabolism	10	6.97E-05	dre00520
	Glycolysis / Gluconeogenesis	12	7.72E-05	dre00010
36hpf	Arachidonic acid metabolism	5	0.000137668	dre00590
	Arginine and proline metabolism	5	0.000408567	dre00330
	Fatty acid elongation	3	0.002138082	dre00062
	Glycine, serine and threonine metabolism	3	0.005393213	dre00260
	Purine metabolism	6	0.032194106	dre00230
	Glycolysis / Gluconeogenesis	3	0.034824781	dre00010
	Vascular smooth muscle contraction	5	0.035029812	dre04270
	Regulation of actin cytoskeleton	7	0.045848576	dre04810
	Peroxisome	3	0.048863853	dre04146
	Lysosome	4	0.067628304	dre04142
60hpf	Phototransduction	9	9.46E-09	dre04744
	Starch and sucrose metabolism	5	0.000134923	dre00500
	Arachidonic acid metabolism	6	0.000192593	dre00590
	Ferroptosis	5	0.00065748	dre04216
	Linoleic acid metabolism	3	0.001400606	dre00591
	Metabolism of xenobiotics by cytochrome P450	4	0.001993945	dre00980
	Pentose and glucuronate interconversions	3	0.001996298	dre00040
	Ether lipid metabolism	4	0.003844184	dre00565
	Glycine, serine and threonine metabolism	4	0.004242522	dre00260
	Adipocytokine signaling pathway	6	0.004444887	dre04920
96hpf	Linoleic acid metabolism	5	2.06E-07	dre00591
	Arachidonic acid metabolism	7	2.65E-07	dre00590
	Steroid biosynthesis	4	4.77E-06	dre00100
	Porphyrin and chlorophyll metabolism	3	0.000775071	dre00860
	Glycerolipid metabolism	4	0.001197725	dre00561
	Metabolism of xenobiotics by cytochrome P450	3	0.001498183	dre00980
	Herpes simplex infection	6	0.010009248	dre05168
	Drug metabolism - other enzymes	3	0.011068023	dre00983
	p53 signaling pathway	3	0.014887859	dre04115
	Cardiac muscle contraction	3	0.037957564	dre04260
